# Species delimitation and recognition in the *Pediomelum megalanthum* complex (Fabaceae) via multivariate morphometrics

**DOI:** 10.3897/phytokeys.44.8750

**Published:** 2015-01-13

**Authors:** Ashley N. Egan

**Affiliations:** 1 Smithsonian Institution, U.S. National Herbarium, National Museum of Natural History, 10th and Constitution Ave, Washington D.C. 20013, USA

**Keywords:** *Pediomelum*, principal component analysis, multivariate morphometrics, species delimitation, cluster analysis, Fabaceae

## Abstract

*Pediomelum* is a genus endemic to North America comprising about 26 species, including the *megalanthum* complex, which consists of *Pediomelum megalanthum* and its varieties *retrorsum* and *megalanthum*, *Pediomelum mephiticum*, and the recently described *Pediomelum verdiense* and *Pediomelum pauperitense*. Historically, species of the *megalanthum* complex have been variably recognized at the species or variety levels, dependent upon the relative importance of morphological characters as diagnostic of species. Ten quantitative morphological characters regarded as diagnostic at the species level were analyzed using multivariate morphometrics across these taxa in order to examine the discriminatory power of these characters to delineate species and to aid in species delimitation. The analyses support the recognition of *Pediomelum megalanthum*, *Pediomelum mephiticum*, and *Pediomelum verdiense* at the species level, *Pediomelum retrorsum* as a variety under *Pediomelum megalanthum*, and suggest the sinking of *Pediomelum pauperitense* into *Pediomelum verdiense*. The findings of the present study help quantify the power of certain characters at delimiting taxa and provide a basis for taxonomic revision of the *Pediomelum megalanthum* complex.

## Introduction

*Pediomelum* Rydb. (Psoraleeae; Leguminosae) includes about 26 species, all native to North America (Rydberg 1919). The genus radiated recently and rapidly, with diversification shifts taking place within the last 2 mya, likely due to the impacts of Pleistocene glaciations (Egan and Crandall 2008a). This recent radiation is illustrated in the shallow branch lengths within phylogenies, relative to related genera within the Psoraleeae tribe, even phylogenies based on a combined dataset of eight DNA markers (Egan and Crandall 2008b). This is also reflected in the disparate taxonomic views among botanists, with several taxa variably recognized at specific or varietal levels. The most taxonomically contested group of species lie within subgenus *Disarticulatum* *sensu* Grimes (1990), which includes 10 species, with most variably restricted to areas of Texas and the deserts of the southwest U.S.

An example of these contrasting taxonomic views can be found within the subspecific classification of *Pediomelum megalanthum* (Wooton & Standl.) Rydb. Some botanists have recognized this as having three varieties (e.g. Grimes 1990; Isely 1998): var. *megalanthum* is found mostly in Uintah, Grand, and San Juan counties down the eastern border of Utah as well as neighboring counties in Colorado; var. *epipsilum* (Barneby) Grimes is endemic to the Dixie Corridor of Kane Co., UT and neighboring Coconino Co., AZ; var. *retrorsum* (Rydb.) Grimes is restricted to Nye, Lincoln, and Clark Co, NV, Mohave, Coconino, and disjunct in Graham Co AZ. Other botanists treat each as a separate species (e.g. Rydberg 1919; Welsh et al. 1993). Grimes (1990) favors varietal ranking based on overlapping qualitative and quantitative morphological characters, with no clear diagnostic features enabling species distinction. Isely (1998) even throws *Pediomelum mephiticum* (S. Watson) Rydb. into the ‘*megalanthum*-*mephiticum*-*epipsilum*’ complex. On the other hand, Welsh et al. (1993) utilized directionality of pedicel and peduncle hairs (*Pediomelum retrorsum* Rydb. vs. *Pediomelum megalanthum*) and absence of pubescence on the upper leaf surface (*Pediomelum epipsilum* (Barneby) S.L.Welsh) as diagnostic characters to maintain their distinctiveness.

Given the narrow distributions of these taxa and the disparate views on the usefulness of certain morphological characters for species delimitation in this group, many might see this as an example of the age old war between lumpers, those who tend to recognize fewer species, often allowing considerable breadth of morphological variation as inherent in species concepts, and splitters, those who split species based on more minute morphological differences, sometimes down to the population level. Hewitt C. Watson eloquently exemplified this war in a letter to Charles Darwin dated 13 August 1855 wherein he wrote “Taking J. D. Hooker & Jordan as representative men for the opposite factions in botany,—‘lumpers & splitters’, the former would reduce the species of vascular plants to three score thousand, or perhaps much fewer;—while Jordan would raise them to three hundred thousand.” (Darwin Correspondence Database, 25 Sept 2014).

Whilst preparing the treatment of *Pediomelum* for the Flora of North America, I was confronted with the question of where I lie on the spectrum of lumpers vs. splitters, for *Pediomelum megalanthum* and its varieties, and in particular, in regards to two recent species described by Welsh and Licher (2010): *Pediomelum pauperitense* S.L.Welsh, Licher, & N.D. Atwood, described based on six collections, and *Pediomelum verdiense* S.L.Welsh & Licher, described from four specimens. These species are said to differ from *Pediomelum mephiticum* in the directionality of pedicel and peduncle hairs as well as the shape of the lateral and upper calyx teeth. The species are said to differ distinctly from each other in the size of pedicels, bracts, seeds, and flower lengths, as well as flower color and peduncle length. They join the ranks of what I refer to as the *Pediomelum megalanthum* complex in subgenus *Disarticulatum*.

The rapid radiation of the genus coupled with the variable recognition of specific or varietal ranking and the contrasting opinions of the relative discriminatory power and usefulness of several morphological characters invites an approach using multivariate morphometric analysis as a means of delimiting species or varieties among contested taxa. This is particularly so in the case of the newly described *Pediomelum pauperitense* and *Pediomelum verdiense*. This work will additionally examine the relative power of certain quantitative morphological characters historically used to delimit species in this group. Here, I aim to objectively delimit species within the *Pediomelum megalanthum* complex using a multivariate morphometric approach, incorporating the morphological diversity of *Pediomelum megalanthum* (vars. *megalanthum* and *retrorsum*), *Pediomelum mephiticum*, *Pediomelum verdiense*, and *Pediomelum pauperitense*. I have chosen to recognize *Pediomelum epipsilum* at the specific level (discussed below) and so it is not included in these analyses.

## Methods

*Plant material* – Twenty-seven herbarium specimens from Utah and Arizona (deposited at ARIZ, ASC, BRY, and US) were chosen for study and tentatively identified as *Pediomelum mephiticum*, Pediomelum megalanthum var. megalanthum, Pediomelum megalanthum var. retrorsum, *Pediomelum pauperitense*, and *Pediomelum verdiense*. A list of the specimens included in the morphometric analyses, with voucher information and origin, are given in Table 1. Effort was made to obtain all identified specimens of *Pediomelum verdiense* and *Pediomelum pauperitense* through a request to ARIZ, ASC, and BRY for such, especially those listed in Welsh and Licher (2010). However, some specimens were not available for loan. Because of this, comparatively few specimens for these taxa were available. So as not to over-weigh the analyses with specimens from *Pediomelum mephiticum* and *Pediomelum megalanthum* and its varieties, and thereby introducing sampling bias, a comparable number of these specimens were used as well, resulting in a set of specimens smaller than that used in some other studies. However, trends were still very visible in these data. Most specimens were collected or annotated by botanists having authority on the genus, including S.L. Welsh, J.W. Grimes, A.N. Egan, M. Licher, and N.D. Atwood, and taxonomic identifications were initially accepted according to the most recent annotation on the specimens. Those recognized at the specific vs. subspecies levels with the same epithet were analyzed together, i.e. *Pediomelum retrorsum* was grouped in analyses with Pediomelum megalanthum var. retrorsum.

**Table 1.** Specimens and their source herbaria used for morphometric analyses. Only first collectors listed. *specimens listed as paratypes by Welsh and Licher (2010).

Pediomelum megalanthum var. megalanthum: (BRY) - Belnap 244, Licher 1915, Welsh 22771, Welsh 27822, Welsh 22787
Pediomelum megalanthum var. retrorsum: (BRY) - Bundy 140, Atwood 4798, Hughes 3
*Pediomelum mephiticum*: (BRY) - Baird 3080, Welsh 23478, Atwood 5148, Egan 126, Neese 16864; (US) - Atwood 3903, Holmgren 3290, Jones 5095, Jones 5064b
*Pediomelum pauperitense*: (BRY) - Higgins 23137*, Atwood 18013
*Pediomelum verdiense*: (ARIZ) - Wojciechowski 212*, Harbison 41.312*, Demaree 43938; (ASC) - Rink 1840*, Licher 2347; (BRY) - Licher 2009, Licher 2015, Licher 2007.

*Characters scored* – Ten quantitative morphological characters (Table 2) were scored. Characters were chosen based on those quantitative traits widely used in flora and monographic works to distinguish species, particularly flower and calyx characters, as well as those characters used by Welsh and Licher (2010) as diagnostic of species. Other studies have used a similar number of characters for morphometric analysis within species complexes with positive information content (e.g. Kaplan and Marhold 2012; Lihová et al. 2004). Characters were scored for between two and five sites per specimen using digital calipers. While there are a few qualitative characters – such as direction of pedicel and peduncle hairs – that are used by some to differentiate taxa (i.e. *Pediomelum megalanthum* varieties), these were not included here. Different researchers have debated the relative utility and importance of directionality of hairs as diagnostic of species or lower-level taxa (see discussion above). I chose explicitly not to include these characters because my focus is on the use of quantitative (continuous) morphological traits with the aim to determine whether quantitative traits can separate taxa along similar lines as some previous researchers do for species vs. subspecies based on the qualitative character of vestiture (e.g. *Pediomelum megalanthum* varieties).

**Table 2.** Morphometric characters used in this study.

Character acronym	Detailed description of the character
flower length	from the base of the calyx to the tip of the banner
calyx length	from the base of the calyx to the tip of the lower calyx tooth
calyx tube	from the base of the calyx to the beginning of the calyx teeth
lower calyx tooth	from point of attachment on calyx to tip
stipules	from point of attachment to tip
petioles	from point of attachment to base of petiolule
leaflets	from point of attachment to petiolule to tip of terminal leaflet
bracts	from point of attachment to tip
peduncle	from point of attachment on stem to base of first pedicel
pedicel	from point of attachment to peduncle to base of calyx

*Multivariate morphometric analyses* – A combination of multivariate analyses and hierarchical clustering were employed to investigate species limits in this group. All statistics were computed in the statistical package JMP v. 11.1.1 (SAS Institute Inc., Cary, NC). As an initial step, correlation coefficients were computed on the total dataset and on each species’ dataset to reveal any highly correlated character pairs that may distort downstream analyses. In addition, departure from a normal distribution for each character within each species was tested using the Shapiro-Wilk goodness of fit test (Shapiro and Wilk 1965).

Morphometric multivariate analyses were conducted on values of individual measurements without averaging across multiple observations per specimen. This was done because of the limited number of specimens available for *Pediomelum pauperitense* and *Pediomelum verdiense*. Use of all observations both within and across specimens will likely provide a better view of the intraspecific variation within a character. This is akin to Pedersen’s (2010) justification for using values measured on each individual plant as opposed to using a population mean.

A hierarchical cluster analysis (HCA) was performed to investigate how specimens would group based on overall morphological similarity using Ward’s minimum variance method with the data standardized by standard deviation (Ward Jr 1963). This method groups specimens by minimizing the increase in the error sum of squares upon each addition of a cluster. Because the use of multiple methods is recommended to ascertain the robustness of clusters (Marhold 2011), UPGMA (unweighted pair-group method using arithmetic averages) with data standardized by standard deviation was also employed (Sokal and Michener 1958).

Principal component analysis (PCA; Sneath and Sokal 1973) was used to delineate patterns of morphological variation across the *Pediomelum megalanthum* complex. This method is a good first tool for investigating overall patterns in morphology as each character is weighted the same. This was first applied to the complete dataset with all characters and species included. For greater resolution among the main groups, PCA was then conducted on two subgroups: i) *Pediomelum mephiticum*, *Pediomelum verdiense*, and *Pediomelum pauperitense*; ii) Pediomelum megalanthum var. megalanthum and Pediomelum megalanthum var. retrorsum.

Canonical discriminant analyses (CAN) were employed to investigate the spread of means across each species group and determine how well the characters (Y) predicted the separation of species based on means. This method measures the distance of each point from the centroid, or multivariate mean, of its group as defined previously by species or subspecies. The distance measure is based on the Mahalanobis distance, which incorporates the variances and covariances between variables. CAN is classically implemented using a linear method, which assumes that Y variables are normally distributed with the same variances and covariances, or a quadratic method in which covariances can be different across groups. Because not all of the character distributions were normal, I employed a regularized, compromise method, which is a mixture between the linear and quadratic methods (Friedman 1989). The regularized method incorporates two parameters: lambda deals with the shrinkage to a common covariance and ranges from 0 = quadratic to 1 = linear; gamma deals with the shrinkage to diagonal and ranges from 0 = no shrinkage to 1 = diagonals only. A low gamma is suggested when variables are correlated. Here I used a lambda proportional to the number of non-normal character distributions by species (λ = 0.8) and a gamma of 0.

Among the multivariate analyses employed here, several have been used by other researchers to determine those variables causally impacting the separation of species. As a variable reduction technique, PCA helps to discern which characters are responsible for grouping individuals. However, it does not assume an underlying causal model. For determining those characters responsible for delineating species, factor analysis may be a more appropriate method as this technique makes the explicit assumption of an underlying causal model (Jolliffe 2005; Suhr 2005). Factor analysis was performed using principal components and the varimax rotation (FAPC) as well as the maximum likelihood framework with varimax rotation (FAML). Similar to PCA, CAN reduces the variable space to those discriminants that are responsible for assigning individuals to previously defined groups, and is often employed to determine those variables with the most discriminatory power (e.g. Koch et al. 2013; Semple and Chmielewski 1987). Stepwise discriminant analysis (SDA) is a method designed to order variables by discriminatory power, adding variables in a stepwise fashion according to the amount of correlation of the variable to the reduced eigenvectors. SDA was compared to results from PCA, CAN, and factor analyses to provide a robust investigation into which of the morphological characters are most informative at delineating species or taxa.

Each analysis was conducted on a series of three data sets or levels: (1) the first dataset included all species, (2) the second dataset includes only data from *Pediomelum mephiticum*, *Pediomelum verdiense*, and *Pediomelum pauperitense* (the MVP group), (3) the third data set includes the *Pediomelum megalanthum* varieties, Pediomelum megalanthum var. megalanthum and Pediomelum megalanthum var. retrorsum. Datasets are thus notated by the type of analysis and the dataset used such as CAN1, HCA2, PCA3, and so on.

## Results

Following Kaplan and Marhold (2012), a cutoff value of 0.90 for the correlation between characters was used to ascertain exclusion of characters from the data analyses. Correlation coefficients did not exceed 0.90 for any character pair within any species, and so all characters were used in subsequent analyses. Only three character pairs across all species had correlation coefficients above 0.8 in either the positive or negative direction: *Pediomelum verdiense* had a correlation coefficient of 0.8775 for calyx:lower calyx tooth; Pediomelum megalanthum var. retrorsum exhibited a negative correlation of -0.8108 for pedicel:bract; *Pediomelum pauperitense* exhibited a correlation of 0.8783 for flower:bract. For all species, the correlation between calyx:lower calyx tooth was between 0.6 and 0.8, an expected result considering the overlapping nature of these characters. However, inclusion of both incorporates the plasticity of calyx morphology into the overall analysis – a key character used for species delimitation – and thus all characters are kept for further analyses.

The vast majority of character distributions fit a normal curve, with three rejecting the null hypothesis of a normal fit only marginally (0.04 < p < 0.05; Pediomelum megalanthum var. retrorsum:lower calyx tooth, *Pediomelum mephiticum*:peduncle & petioles), three rejecting moderately (0.02 < p < 0.04; leaflets for *Pediomelum verdiense* & *Pediomelum pauperitense*), three rejecting strongly (0.001 < p < 0.01; *Pediomelum pauperitense*:calyx tube*, Pediomelum megalanthum var. megalanthum:flowers & petioles*), and one rejecting very strongly (p=0.0003; *Pediomelum mephiticum*:pedicel*), those distribution with outliers detected are notated with an asterisk. Summary statistics for each character by species are given in Table 3.

**Table 3.** Summary statistics for each character by species. number of observations (n), minimum (min) and maximum (max) values, mean, standard deviation (st. dev.), and 5^th^ and 95^th^ percentiles.

Taxon	Parameter	flower	calyx	calyx tube	lower calyx tooth	stipules	petiole	leaflets	bracts	peduncle	pedicel
*Pediomelum megalanthum*	Min–max	12–18	11.8–16.2	4.8–8.1	6.5–10.2	6.8–12.4	44–150	16–33	5.7–13	8.5–80	3–6.2
var. *megalanthum*	Mean/St.dev.	16/1.716	13.81/1.213	6.28/0.802	8.08/1.103	9.57/1.538	72.05/23.125	25.53/5.265	9.34/1.930	34.15/21.185	4.07/0.896
n=20	5-95 Quantiles	12.03–17.98	11.81–16.16	4.81–8.07	6.5–10.19	6.81–12.38	44.1–147.25	16.15–32.95	5.74–12.98	8.59–79.5	3–6.17
*Pediomelum mephiticum*	Min–max	9.4–12.6	8.2–11.7	2.6–4.1	5.3–8.3	7.4–14	32–120	15–35	6.8–12.5	19.7–65	2.3–5.7
n=34	Mean/St.dev.	10.94/0.583	10.12/0.798	3.52/0.399	6.74/0.734	9.69/1.427	64.76/23.745	24.36/5.195	10.1/1.428	40.64/13.099	3.09/0.734
	5-95 Quantiles	9.93–12.15	8.43–11.48	2.75–4.1	5.45–8.15	7.85–12.58	32.75–113.25	16.5–34.25	7.78–12.43	20.68–64.25	2.3–4.8
*Pediomelum pauperitense*	Min–max	10.7–12.2	9.8–12.1	4.2–5.5	5.5–7.9	8.7–13	57–98	14–28	3.6–7.3	17–54	3–4.2
n=10	Mean/St.dev.	11.53/0.585	10.84/0.869	4.59/0.465	6.49/0.791	10.54/1.377	71.7/14.345	19.6/5.211	5.83/1.218	33.7/11.814	3.62/0.405
	5-95 Quantiles	10.7–12.2	9.8–12.1	4.2–5.5	5.5–7.9	8.7–13	57–98	14–28	3.6–7.3	17–54	3–4.2
*Pediomelum megalanthum*	Min–max	14.8–19.8	12.2–16.2	6.2–7.7	6.4–9.6	7.6–12.9	40–94	18–29	6.8–12.3	19–62	3–5.1
var. *retrorsum*	Mean/St.dev.	17.78/1.402	14.45/1.19	6.94/0.533	7.69/1.154	10.2/1.818	65.42/15.785	22.23/3.265	9.53/1.745	38.4/15.995	3.82/0.667
n=12	5-95 Quantiles	14.8–19.8	12.2–16.2	6.2–7.7	6.4–9.6	7.6–12.9	40–94	18–29	6.8–12.3	19–62	3–5.1
*Pediomelum verdiense*	Min–max	10.3–15	8.5–13	3–4.9	4.5–9	6–11.7	22–95	14–30	5–10	11–35	2.3–6
n=34	Mean/St.dev.	12.2/0.983	10.82/1.216	4.09/0.412	6.88/1.239	8.9/1.37	52.63/15.876	20.75/4.465	7.13/1.092	22.53/7.184	3.93/0.912
	5-95 Quantiles	10.43–14.03	8.7–13	3.2–4.84	4.89–9	6.13–11.7	23.95–84.6	14.65–30	5.46–9.35	11.65–34.35	2.56–6

**Figure 1.**
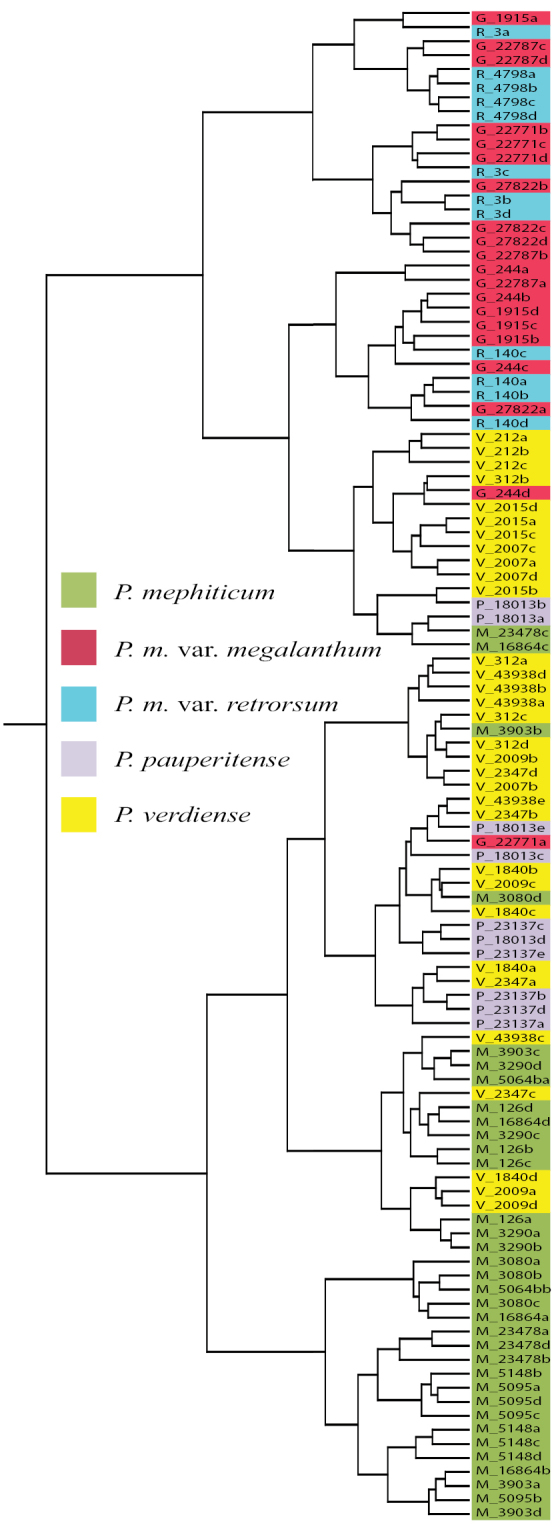
Dendrogram based on all characters for all specimens using Ward’s cluster analysis.

**Figure 2.**
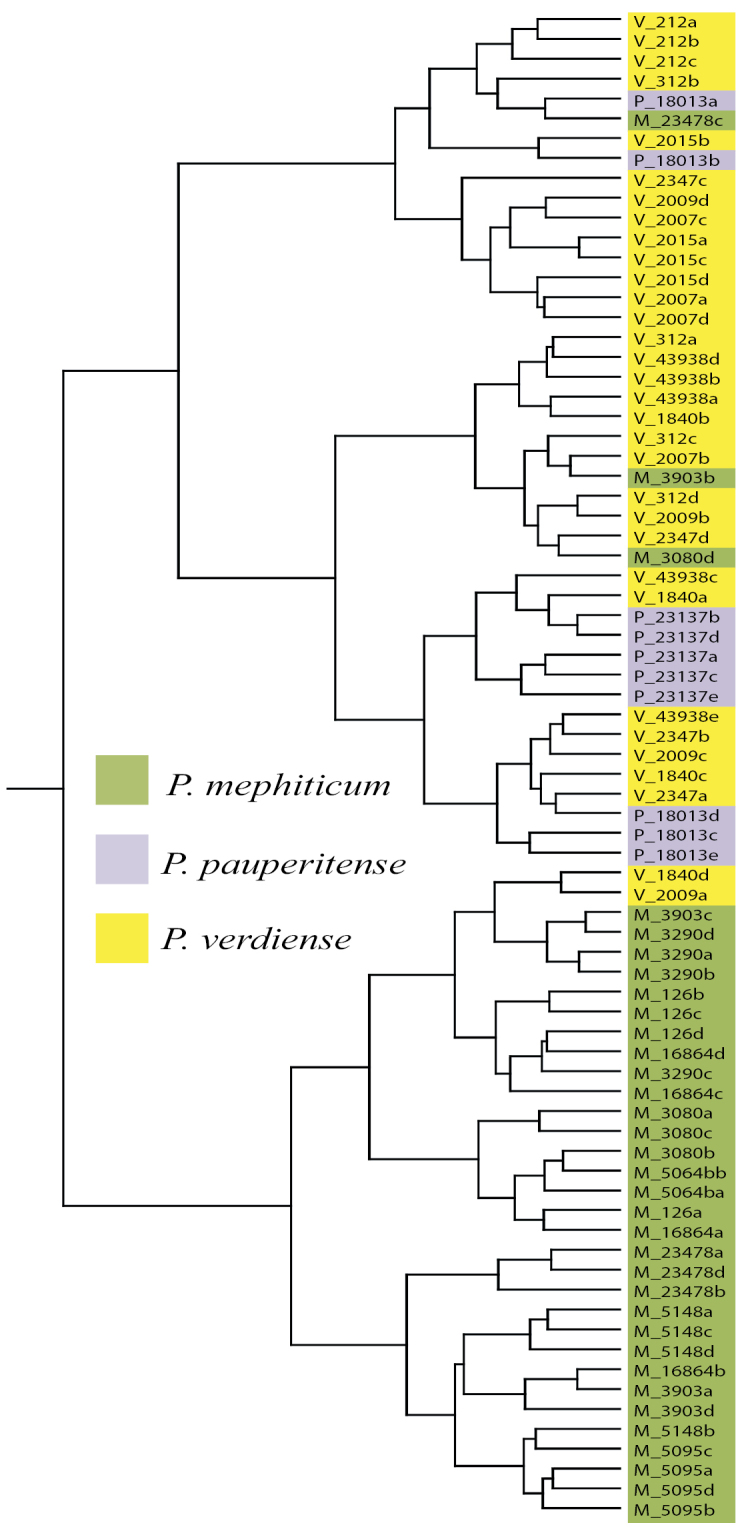
Dendrogram using Ward’s cluster analysis for *Pediomelum mephiticum*, *Pediomelum verdiense*, and *Pediomelum pauperitense*.

Ward’s cluster analysis of all specimens (HCA1) produced a dendrogram with two main groups: one comprised of entirely *Pediomelum mephiticum*, *Pediomelum verdiense*, and *Pediomelum pauperitense* with the exception of a single Pediomelum megalanthum var. megalanthum data point, and the other group comprised of three subgroups, two comprising mixtures of the two *Pediomelum megalanthum* varieties and one comprised mainly of *Pediomelum verdiense* (Fig. 1). UPGMA cluster analysis (data not shown) produced three main clusters, two comprised of a mixture of *Pediomelum megalanthum* varieties, one with a few *Pediomelum verdiense* or *Pediomelum mephiticum* specimens included, and one comprised wholly of the *Pediomelum mephiticum*-*verdiense*-*pauperitense* complex (hereafter denoted as the MVP group), again with exception of the single Pediomelum megalanthum var. megalanthum data point. Considering the strong support for the MVP group, a separate hierarchical analysis was conducted on the MVP group only (HCA2). This analysis suggests two main clusters, one cluster almost entirely of *Pediomelum mephiticum* data points with two *Pediomelum verdiense* data points included therein, and a second main cluster mostly comprised of *Pediomelum verdiense* and *Pediomelum pauperitense* with three *Pediomelum mephiticum* data points scattered throughout (Fig. 2). A cluster analysis of the *Pediomelum megalanthum* varieties (HCA3) showed no clear division between taxa (data not shown).

**Figure 3.**
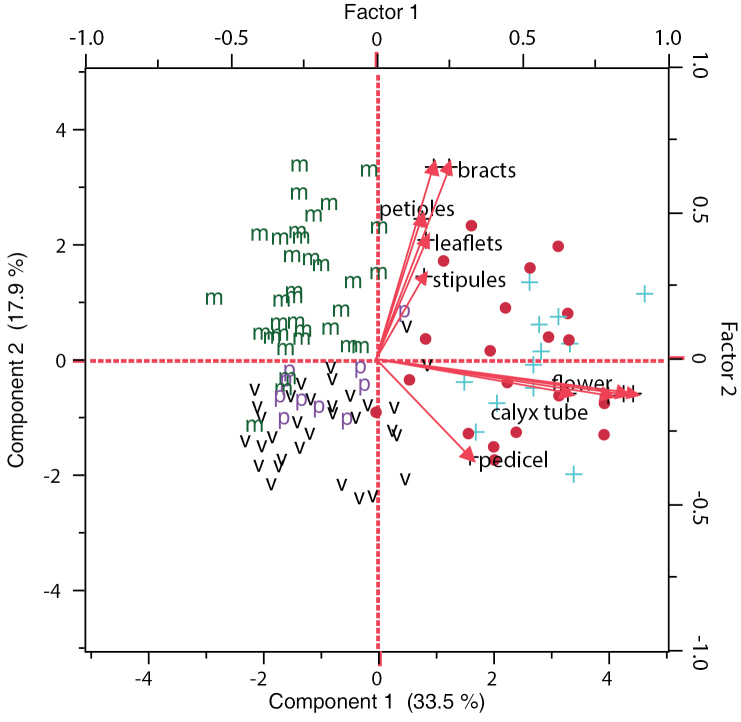
Principal component analysis ordination diagram incorporating all specimens with symbols according to initial identifications. **m** *Pediomelum mephiticum*, **v** *Pediomelum verdiense*, **p** *Pediomelum pauperitense*, ● Pediomelum megalanthum var. megalanthum, + Pediomelum megalanthum var. retrorsum. Principal component scale on the left and bottom; unrotated factor loading scale on the top and right. Exact loading matrix vector lengths are listed in Table 5.

The ordination diagram from the principal component analysis based on all specimens (PCA1; Fig. 3) also suggested two main groups. Specimens of *Pediomelum mephiticum*, *Pediomelum verdiense*, and *Pediomelum pauperitense* were separated from the *Pediomelum megalanthum* varieties along the first axis with all characters contributing to this division. The second axis separated *Pediomelum mephiticum* from a mixture of *Pediomelum verdiense* and *Pediomelum pauperitense* but did not separate the *Pediomelum megalanthum* varieties from each other. Floral characters (flower, calyx, calyx tube, lower calyx tooth, pedicel) vs. vegetative characters (peduncle, petiole, bracts, stipules, and leaflets) separated *Pediomelum verdiense* and *Pediomelum pauperitense* from *Pediomelum mephiticum* along the second axis, with vegetative characters contributing more to differentiation along the second component (Fig. 3; Table 5).

**Figure 4.**
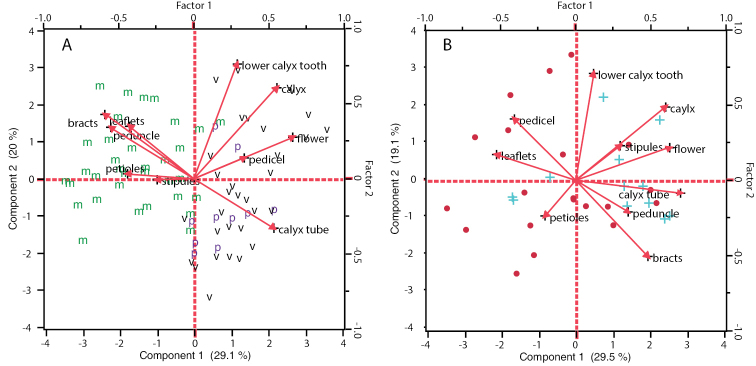
Principal component analysis ordination diagram for **A**) PCA2 and **B**) PCA3. **m** *Pediomelum mephiticum*, **v** *Pediomelum verdiense*, **p** *Pediomelum pauperitense*, ● Pediomelum megalanthum var. megalanthum, + Pediomelum megalanthum var. retrorsum. Principal component scale on the left and bottom; unrotated factor loading scale on the top and right. Exact loading matrix vector lengths are listed in Table 5.

Independent principal component analyses were also conducted on the two main subgroups defined by PCA1. PCA2, comprising the MVP group, showed good separation along the first axis of *Pediomelum mephiticum* from *Pediomelum verdiense* and *Pediomelum pauperitense*, with floral vs. vegetative characters strongly affiliated with this break (Fig. 4A). However, *Pediomelum verdiense* and *Pediomelum pauperitense* created a mixed group with most of the *Pediomelum pauperitense* data points clustering below the second axis (capturing 20% of the variation in the data) amidst *Pediomelum verdiense*, suggestive of a lack of differentiation normally found between species. The same result is found in PCA3, the analysis of the differentiation between Pediomelum megalanthum var. megalanthum and Pediomelum megalanthum var. retrorsum, illustrating a mixture of Pediomelum megalanthum var. megalanthum and Pediomelum megalanthum var. retrorsum data points (Fig. 4B). Contributions of characters to each multivariate axis for each of the three PCA analyses are listed in Table 4.

**Table 4.** Eigenvector contributions (PC) of each character from the first two axes of the principal component analyses based on all morphological characters incorporating all species (PCA1), the *Pediomelum mephiticum*-*Pediomelum verdiense*-*Pediomelum pauperitense* (MVP) complex only (PCA2), and for the two varieties of *Pediomelum megalanthum* (PCA3). Characters are described in Table 2.

	PCA1 (Fig. 3)		PCA2 (Fig. 4A)		PCA3 (Fig. 4B)	
**Character**	**PC1**	**PC2**	**PC1**	**PC2**	**PC1**	**PC2**
**flower**	0.502	-0.098	0.432	0.218	0.424	0.186
**calyx**	0.522	-0.095	0.362	0.484	0.405	0.413
**calyx tube**	0.472	-0.101	0.350	-0.262	0.473	-0.072
**lower calyx tooth**	0.390	-0.094	0.189	0.608	0.082	0.600
**stipules**	0.097	0.229	-0.162	-0.004	0.201	0.195
**petioles**	0.092	0.391	-0.292	0.024	-0.137	-0.198
**leaflets**	0.103	0.330	-0.282	0.277	-0.360	0.145
**bracts**	0.150	0.536	-0.389	0.342	0.324	-0.426
**peduncle**	0.116	0.536	-0.366	0.270	0.235	-0.176
**pedicel**	0.191	-0.272	0.220	0.111	-0.278	0.345

**Figure 5.**
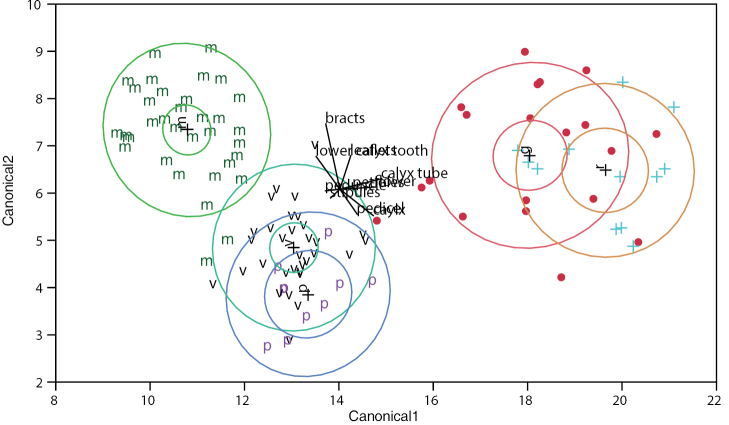
Canonical plot of points and means from linear discriminant analysis of all species by all characters with groups defined as: **m** *Pediomelum mephiticum*, **v** *Pediomelum verdiense*, **p** *Pediomelum pauperitense*, ● Pediomelum megalanthum var. megalanthum, + Pediomelum megalanthum var. retrorsum. Inner circles by group are the 95% confidence region for containing the true overall mean of the group; the outer circles by group are the normal 50% contours, the normal ellipse region that contains 50% of the population for each group. Rays show the coordinate directions in canonical space.

Canonical and classificatory discriminant analyses were also conducted to investigate the spread of means per species group. In accordance with HCA and PCA analyses, CAN1 shows *Pediomelum mephiticum* largely distinct from the others, with the two *Pediomelum megalanthum* taxa creating one group while *Pediomelum verdiense* and *Pediomelum pauperitense* another (Fig. 5). The inner circles of each species represent the 95% confidence region for the true mean (of all characters taken together) of the species. The 95% confidence region for *Pediomelum verdiense* and *Pediomelum pauperitense* are overlapping, as are those of Pediomelum megalanthum var. megalanthum and Pediomelum megalanthum var. retrorsum, suggesting that the overall mean for these species based on all characters is not statistically different.

**Figure 6.**
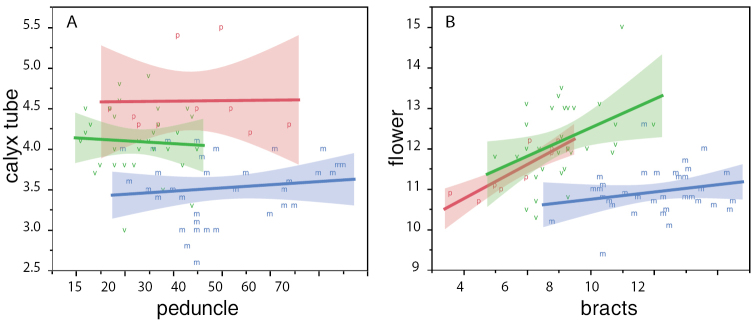
Scatterplot and linear regression of key distinguishing character traits for the MVP group. Fit of **A**) peduncle by calyx tube and **B**) bracts by flower sizes for *Pediomelum mephiticum* (m, blue color), *Pediomelum verdiense* (v, green color), and *Pediomelum pauperitense* (p, red color). All measurements in mm. Line is regression by taxon with accompanying 95% confidence band shaded.

Factor analyses and PCA were taken together to help elucidate those characters most influential in separating predefined groups based on expert identification (Table 5). For the all species dataset, FAPC1, FAML1, and PCA1 all suggested that the separation of MVP from the *Pediomelum megalanthum* taxa calculated along the first factor or component are most highly influenced by floral traits (flower, calyx, calyx tube, and lower calyx tooth). The second factor or component axis largely separates *Pediomelum mephiticum* from *Pediomelum verdiense* and *Pediomelum pauperitense* and is most strongly associated with various vegetative characters (Table 5). This same association between discriminatory power, axes, and characters follows into analyses conducted on the MVP group only (FAPC2, FAML2, and PCA2), but with less distinction between the relative discriminatory power for vegetative vs. floral traits. Analyses of only *Pediomelum megalanthum* varieties showed no clear pattern between floral or vegetative traits as being most discriminatory, but showed several associations that differed across analytical method (Fig. 5). Stepwise discriminatory analysis showed flower length to be the only significant trait offering some measure of discriminatory power between Pediomelum megalanthum var. megalanthum and Pediomelum megalanthum var. retrorsum (SDA3; Table 6) with all other characters not significant. For MVP only, SDA2 determined that bracts were the most important factor in discriminating between species, followed by flower length, pedicel length, peduncle, calyx tube, and stipules (Table 6). Scatterplots showing the fit of peduncle × calyx tube lengths (Fig. 6A) and bract × flower lengths (Fig. 6B) show the range and distinguishing power of these traits for MVP. For all species analyzed together, SDA1 calyx tube length was strongly correlated with separation of groups, followed by bract and flower lengths; leaflets, pedicel, peduncle, and stipules were also associated with discriminatory power across these groups, but less so (Table 6).

**Table 5.** Relative distinguishing power of characters between species by factor or PCA analyses. Bold factors are those highly associated with the corresponding loading.

	FAPC-rotated loadings		FAML-rotated loadings			PCA-loading matrix	
All specimens	FA1 (32.2%)	FA2 (19.3%)	FA1 (26.6%)	FA2 (12.8%)	FA3 (12.5%)	PC1 (33.5%)	PC2 (17.9%)
	mvp|gr	m|vp	mvp|gr	m|vp	m|vp	mvp|gr	m|vp
flower	**0.917418**	0.146258	**0.951893**	0.091466	0.02346	**0.91974**	-0.13088
calyx	**0.951527**	0.160732	**0.914948**	0.135937	0.192597	**0.9566**	-0.12712
calyx tube	**0.865426**	0.125783	**0.838602**	0.109935	**0.475039**	**0.86402**	-0.13511
lower tooth	**0.718706**	0.090375	0.243157	-0.207947	0.216766	**0.71338**	-0.12566
stipules	0.078849	0.345099	0.044324	**0.807081**	0.096593	0.17714	0.30648
petioles	0.006661	**0.549323**	0.003746	**0.629476**	0.071232	0.16841	**0.52291**
leaflets	0.04924	**0.477942**	0.087371	0.278027	-0.074971	0.18804	**0.44215**
bracts	0.051288	**0.765723**	-0.028996	0.236017	0.206967	0.27489	**0.71652**
peduncle	-0.008032	**0.748081**	0.125539	0.146123	-0.014782	0.21301	**0.71716**
pedicel	**0.440783**	-0.24418	0.357629	0.015441	**0.933736**	0.34914	-0.36334
**MVP only**	**FA1 (25.5%)**	**FA2 (23.7%)**	**FA1 (20.3%)**	**FA2 (19%)**	**FA3 (12.9%)**	**PC1 (29.1%)**	**PC2 (20%)**
	**m|vp**	**vp|v**	**m|vp**	**m~vp**	**p~v**	**m|vp**	**vp|v**
flower	-0.374642	**0.705445**	**0.991576**	0.090467	-0.092824	**0.73706**	0.30781
calyx	-0.044509	**0.920401**	**0.783954**	-0.304062	-0.105493	**0.61815**	**0.68338**
calyx tube	**-0.697581**	0.092945	**0.574663**	0.254593	-0.285595	**0.59828**	-0.37056
lower tooth	0.29626	**0.868977**	0.206384	0.173869	0.009934	0.32207	**0.85975**
stipules	0.209798	-0.179713	0.176519	**0.973894**	0.144094	-0.27618	-0.00589
petioles	**0.407106**	-0.289188	0.046761	-0.393911	**0.898281**	**-0.49816**	0.03461
leaflets	**0.620859**	-0.002673	-0.131126	-0.151598	**0.410291**	**-0.48171**	0.3917
bracts	**0.819303**	-0.046928	-0.038605	**-0.658242**	0.367456	**-0.66321**	**0.48334**
peduncle	**0.724966**	-0.100322	-0.054292	0.073884	0.210615	**-0.62414**	0.38222
pedicel	-0.190591	0.359369	0.027441	-0.371378	0.117906	0.37528	0.15697
***Pediomelum megalanthum* varieties**	**FA1 (25.2%)**	**FA2 (23.4%)**	**FA1 (21%)**	**FA2 (19%)**		**PC1 (29.5%)**	**PC2 (19.1%)**
	**g~r**	**gr**	**g~r**	**gr**		**g~r**	**gr**
flower	0.392436	**0.664925**	**0.745911**	0.264933		**0.7282**	0.25664
calyx	0.16657	**0.885112**	**0.614819**	-0.08418		**0.69691**	**0.57051**
calyx tube	**0.68603**	**0.446127**	**0.535314**	0.386092		**0.81223**	-0.09975
lower tooth	**-0.425283**	**0.725083**	0.318941	0.092851		0.14083	**0.82872**
stipules	0.091589	**0.427999**	-0.043631	-0.156071		0.34545	0.26877
petioles	-0.003729	-0.36035	0.279887	**0.927344**		-0.23466	-0.27349
leaflets	**-0.602025**	-0.244695	-0.361722	**0.823352**		**-0.61834**	0.19993
bracts	**0.805107**	-0.091753	0.188143	0.265238		**0.55739**	**-0.58816**
peduncle	**0.465463**	0.074153	**-0.493267**	-0.003841		**0.40407**	-0.24265
pedicel	**-0.672394**	0.057123	**-0.538243**	-0.170644		**-0.47806**	**0.47628**

FAPC, factor analysis on principal components. FAML, factor analysis via maximum likelihood. PCA, principal component analysis. FA1, factor 1. FA2, factor 2. FA3, factor 3. PC1, principal component 1. PC2, principal component 2. (%), amount of variance explained by that factor or component. m, *Pediomelum mephiticum*. v, *Pediomelum verdiense*. p, *Pediomelum pauperitense*. g, Pediomelum megalanthum var. megalanthum. r, Pediomelum megalanthum var. retrorsum. Symbols signify a strong (|) or moderate (~) split between indicated taxa.

**Table 6.** Rank order of relative discriminatory power of characters for distinguishing species as ascertained by stepwise discriminatory analysis. F-ratio and probability are calculated based on the stepwise inclusion into the set of characters ranked previously.

	All specimens			MVP only			*Pediomelum megalanthum* varieties		
rank	Character	F-ratio	Prob>F	Character	F-ratio	Prob>F	Character	F-ratio	Prob>F
1	**calyx tube**	156.911	0.0000	**bracts**	66.759	0.0000	**flower**	9.19	0.0050
2	**bracts**	28.938	0.0000	**flower**	25.432	0.0000	leaflets	2.32	0.1385
3	**flower**	13.322	0.0000	**pedicel**	12.814	0.0000	lower tooth	1.711	0.2015
4	**leaflets**	5.107	0.0009	**peduncle**	10.466	0.0001	bracts	1.299	0.2643
5	**pedicel**	3.527	0.0098	**calyx tube**	4.649	0.0128	calyx	1.957	0.1737
6	**peduncle**	3.769	0.0068	**stipules**	3.539	0.0345	petioles	0.144	0.7072
7	**stipules**	3.497	0.0103	petioles	1.543	0.2213	peduncle	0.062	0.8055
8	petioles	1.681	0.1607	leaflets	1.339	0.2690	calyx tube	0.052	0.8215
9	lower tooth	1.445	0.2252	calyx	1.475	0.2363	stipules	0.009	0.9257
10	calyx	2.015	0.0986	lower tooth	0.722	0.4897	pedicel	0	0.9864

MVP, the dataset including only *Pediomelum mephiticum*, *Pediomelum verdiense*, and *Pediomelum pauperitense*. Bold characters are those showing significant discriminatory power as ranked in the inclusion set.

## Discussion

Species recognition often relies on deciphering nonoverlapping patterns in morphology between biological entities (Davis and Heywood 1963; Mayr 1942), with these gaps in morphology used as a means of delimiting species or lower taxa. Recognition of taxa at the specific vs. varietal or subspecific level can often be a difficult choice to make, especially for plants. Ideally, subspecies or varieties should be characterized by some cohesive trait along side morphology, such as geography, ecology, or phylogenetic traits (Hamilton and Reichard 1992). Often this is exacerbated by a disagreement concerning the relative importance of various morphological characters as being diagnostic of species or varieties or the lack of a clear supporting character not of the morphological type. This battle is evident in the genus *Pediomelum*, particularly for species of the intermountain west.

Relationships among the species or varieties of the *Pediomelum megalanthum* complex have been debated among botanists, largely due to differing opinions as to which morphological characters are most important for distinguishing species. In his key to species of *Pediomelum*, Rydberg (1919) emphasized the pubescence of the peduncle (hairs appressed vs. hairs spreading or retrorse) as diagnostic between *Pediomelum megalanthum* and *Pediomelum mephiticum* or *Pediomelum retrorsum*. Welsh et al. (1993) followed Rydberg (1919). Ockendon (1965) commented extensively on the *Pediomelum megalanthum* complex, suggesting that this group of species required an extensive review coupled with reproductive studies to truly decipher amongst species. He considered the pubescence character to be useful, but recognized that use of pubescence on the peduncle could be problematic, favoring that of the petioles as being more diagnostic and leading to his recognition of *Psoralea megalantha* Wooton & Standl. and *Psoralea mephitica* S.Watson, with specimens relegated to *retrorsum* being subsumed under *Psoralea mephitica*. Ockendon (1965) also recognized the potential of flower size as a distinguishing character and suggested that new species might be recognized in the future. Ultimately, Ockendon (1965) concluded that “Rather than altering the taxonomy of this group in a piecemeal fashion, it seems best to wait until a more unified treatment is possible.”

Grimes (1990) took up Ockendon’s challenge and produced the most recent treatment of the genus, taking a more quantitative approach emphasizing flower and calyx lengths. This relative increase in the importance of flower size as delimiting between taxa in this species group admitted less variability in both *Pediomelum mephiticum* and *Pediomelum megalanthum*, these being distinguished by those plants having calyx tubes 4.5 mm or shorter vs. those 6 mm or longer, respectively. This shifted specimens previously referred by Ockendon (1965) to *Psoralea mephitica* being now recognized as a variety of *Pediomelum megalanthum* due to flower size and other overlapping quantitative characters (see Table 5 of Grimes 1990).

The morphometric analyses conducted herein largely support the use of flower and calyx sizes as being useful characters for species delimitation. All level 1 morphometric analyses involving all species in the complex, *Pediomelum mephiticum*, *Pediomelum verdiense*, *Pediomelum pauperitense*, and the two varieties of *Pediomelum megalanthum*, illustrated a clean break in overall morphological variation between *Pediomelum mephiticum*, *Pediomelum verdiense*, and *Pediomelum pauperitense* (the MVP group) and the *Pediomelum megalanthum* varieties (HCA1, PCA1; Figs 1, 3). Canonical discriminant analysis (CAN1; Fig. 5) also illustrated a strong break between the MVP group and the *Pediomelum megalanthum* varieties, with no overlap in overall species means. Flower size, calyx, and calyx tube were most strongly associated with the first canonical axis, while calyx tube and bracts showed the strongest association with the second canonical axis.

In most analyses, floral characters separated species with the greatest discriminatory power along the first component or axis of the analysis, whereas the suite of vegetative characters contributed more to the second component divisions (Fig. 3; Table 5). These results are not unexpected, as most current species determinations were based on Grimes’ (1990) criteria of flower and calyx morphology as being most distinguishing of species. The stepwise discriminatory analysis for all taxa (SDA1) suggested that the strongest discriminatory character amongst taxa was calyx tube length (Table 6), a finding in line with most researchers who use this character in dichotomous keys to separate species (Grimes 1990; Isely 1998; Welsh et al. 1993) and is here largely responsible for the separation of the *megalanthum* varieties from the MVP group. Bract size is the second most discriminatory character (Table 6), responsible largely for the separation of *Pediomelum mephiticum* from *Pediomelum verdiense* + *Pediomelum pauperitense*. Flower size is the third most discriminating character for distinguishing amongst the previously determined species groups.

The distinction of large and small flowered forms within the *megalanthum* complex has been recognized by previous researchers (Grimes 1990; Isely 1998). The larger-flowered group comprising the *Pediomelum megalanthum* varieties has been another battleground for taxonomists in this group. Pediomelum megalanthum var. retrorsum has variably been recognized at the varietal level (Grimes 1990; Isely 1998), at the specific level as *Pediomelum retrorsum* (Rydberg 1919; Welsh et al. 1993), or included as either *Psoralea megalantha* or *Psoralea mephitica* (Rydberg 1919), depending on the relative importance of flower size vs. pubescence type and direction of peduncular and petiolar hairs deemed by the researcher. That said, the analyses herein do not support a clear distinction between species designated as *Pediomelum retrorsum* vs. *Pediomelum megalanthum* based on the quantitative characters employed, by either principal component analyses (PCA1, PCA3; Figs 3, 4B) or canonical discriminant analysis (CAN1; Fig. 5). In fact, all of the ten characters employed here present overlapping character ranges (Table 3), a finding illustrated best by the canonical discriminant analysis that shows strong overlap in the 50% normal contours, the normal ellipse region (outer circle) that contains 50% of the species’ quantitative morphological diversity based on the 10 characters included here (Fig. 5). In addition, there is an overlap, albeit slight, in the 95% confidence region between *megalanthum* and *retrorsum*, suggesting that the overall morphological means are not statistically different. Grimes (1990) presented a comparison of 14 quantitative characters across the three varieties of *Pediomelum megalanthum* and concluded something very similar to these findings: “…the varieties overlap in most qualitative and quantitative characters, and the diagnostic characters for all three varieties overlap so much as to make specific status untenable.” (see Table V of Grimes 1990: 81).

That said, there is some quantitative morphological and geographic separation evident between Pediomelum megalanthum var. megalanthum and Pediomelum megalanthum var. retrorsum. The varieties are fairly distinct geographically, with *megalanthum* primarily of eastern Utah, western Colorado, and northwest New Mexico and *retrorsum* of southern Nevada, northwestern Arizona, and sporadically along the Gila River drainage elsewhere in Arizona. Flower length was the only character having significant discriminatory power in the stepwise discriminant analysis (SDA3; Table 6), suggesting that flower length may be the only causal quantitative character offsetting the separation of the population normal contours in the canonical discriminant analysis. This suggests that perhaps there is ongoing differentiation among the *megalanthum* varieties, perhaps spurred by geographic separation that may in time lead to species differentiation. The lack of quantitative separation from each other argues against recognizing these taxa as separate varieties and instead lumping them under *Pediomelum megalanthum*. However, some qualitative difference in the directionality of prevailing hair types and geographic separation exists, providing some justification for recognition and separation at the varietal level. In an effort to favor tradition and lessen the upset to prevailing taxonomic concepts, I am in favor of recognizing these taxa at the varietal level as Pediomelum megalanthum var. megalanthum and Pediomelum megalanthum var. retrorsum, largely following the concept of Grimes (1990).

I agree with Grimes’ conclusions to a point with the recognition of Pediomelum megalanthum var. megalanthum and Pediomelum megalanthum var. retrorsum. However, after careful comparison of the character ranges of Pediomelum megalanthum var. epipsilum with the others as ascertained by Grimes, I find sufficient distinguishing characters that separate *Pediomelum epipsilum* from the other varieties, including having leaflets smaller and glabrate above or sparingly strigose along veins (vs. leaflets larger and pubescent above and below in vars. *megalanthum* and *retrorsum*), and bracts larger and caudate (vs. smaller, acuminate, acute, or shortly caudate in the others). Indeed, Grimes’ quantitative character comparison shows non-overlapping ranges for leaflet and bract size, separating *Pediomelum epipsilum* from the others. Barneby (1943), in his description of the species as *Psoralea epipsila* Barneby, states that it differs from *Psoralea mephitica* by its caulescent nature and conspicuous bicolored leaves and that it is intermediate between *Psoralea mephitica* and its variety Psoralea megalanthum var. retrorsa (Rydb.) Kearney & Peebles in flower size. He also states that the banner is barely exserted from the calyx, whereas the others are well exserted. This preponderance of both quantitative and qualitative differences, coupled with the fact that *Pediomelum epipsilum* is set apart in phylogenetic analyses (Egan and Crandall 2008b), lead me to recognize *Pediomelum epipsilum* at the species level, as others before have also done (Barneby 1943; Welsh et al. 1993).

During an examination of *Pediomelum* in Arizona, Welsh and Licher (2010) discovered some collections that did not key out well based on flower size and peduncular pubescence. All these offending specimens had small flowers and ascending hairs on pedicels and peduncles, a character combination at odds with the prevailing concepts of *Pediomelum megalanthum* and *Pediomelum mephiticum*. Those plants having flowers with a banner that is purple or white suffused with purple that is not strongly contrasting with the wings or keel in color and found mainly from the Verde Limestone Formation were described as *Pediomelum verdiense* whilst those plants from near Poverty Mountain having a banner and wings of white or cream that contrasts strongly to the purple color of the keel, with leaves that tend to exceed inflorescence in height were described as *Pediomelum pauperitense*. For comparison, *Pediomelum mephiticum* has a white or cream banner with wings and keel purple or white suffused with purple and is present in the extreme northwest corner of Arizona and adjacent areas in Utah and Nevada.

Furthermore, Welsh and Licher (2010) stated that *Pediomelum verdiense* corresponded to those plants with pedicels 3–3.5(–5) mm long, bracts 5–8 mm long; flowers 10–11.3 mm long whereas *Pediomelum pauperitense* corresponded to those plants with pedicels 1.5–2.5(–3) mm long, bracts 3–5 mm long; and flowers 7.3–10 mm long. These ranges suggest a clean break between these species and were used to key out specimens. However, my examination and measuring of specimens used in this study, many of which were also cited by Welsh and Licher or were paratypes thereto, gave a very different picture (Table 3). In fact, of these characters, my measurements for *Pediomelum pauperitense* pedicel and flower length were completely non-overlapping with the ranges suggested by Welsh and Licher (pedicels 3–4 mm and flowers 10.7–12.2, as compared to those above). This made me question the authenticity of these species.

The multivariate morphometric analyses on the MVP group only (level 2 analyses) were very telling. There seems to be a distinct separation between *Pediomelum mephiticum* and the other two species, as evidenced by all methods applied herein, but strong overlap between *Pediomelum verdiense* and *Pediomelum pauperitense*. This is perhaps best illustrated by the hierarchical cluster analysis (HCA2) which shows two main clusters, one cluster almost entirely of *Pediomelum mephiticum* and a second main cluster mostly comprised of *Pediomelum verdiense* and *Pediomelum pauperitense* (Fig. 2).

As in the case with the *Pediomelum megalanthum* varieties, canonical discriminant analysis (Fig. 5) shows strong overlap in the 50% normal contours as well as a large overlap in the 95% confidence region between *Pediomelum verdiense* and *Pediomelum pauperitense*, suggesting that the overall morphological means are not statistically different. Lastly, principal component analyses (Figs 3, 4A) showed separation of *Pediomelum mephiticum* from the others along the first component. Factor analysis suggested that flower, calyx tube, peduncle and bracts contributed most to the separation along the first axis. A linear regression of peduncle vs. calyx tube (Fig. 6A) and of bracts vs. flower (Fig. 6B) supports the distinction between *Pediomelum mephiticum* and *Pediomelum verdiense*+*Pediomelum pauperitense* and illustrates the lack of distinction between the latter two taxa. This is evident in the overlapping 95% confidence bands between *Pediomelum verdiense* and *Pediomelum pauperitense* in both linear regressions, with no overlap with *Pediomelum mephiticum*. SDA2 suggests that bracts are the most distinguishing character in the MVP group, followed by flower, pedicel, peduncle and calyx tube in rank order (Table 6).

Given the overlap in continuous character distributions between several species or taxa in this study, some researchers may invoke hybridization as one reason behind overlapping morphology. Traditionally, hybridization is said to create morphological intermediacy (Anderson 1949). However, several studies have shown that hybridization does not always result in morphological intermediacy, but that it can, in fact, produce parental and even novel morphological characters or combinations (e.g. Rieseberg 1995; Rieseberg and Ellstrand 1993). Furthermore, the use of multivariate morphometric techniques for detecting hybridization has been called into question, as these methods cannot distinguish between divergence and hybridization (Wilson 1992). With these caveats in mind, the presence of hybridization within or between taxa in this complex cannot be proven nor ruled out. Indeed, it is possible that *Pediomelum verdiense* or *Pediomelum pauperitense*, or any of the species in this group, could be hybrids involving one or more of the other species in the complex. However, this study cannot address this at this time. The role of hybridization in this complex may best be addressed using molecular or genomic methods spanning the species and population levels.

Taken together, the results of this study argue for the recognition of *Pediomelum mephiticum* and *Pediomelum verdiense* at the specific level, but do not support *Pediomelum pauperitense* as its own species. Some researchers might suggest that *Pediomelum pauperitense* be recognized as a variety of *Pediomelum verdiense* based on geographic separation, differences in peduncle length relative to petiole length, or flower color. However, given the few numbers of populations and specimens relegated to *Pediomelum verdiense* and *Pediomelum pauperitense*, I deem it premature to make this distinction, especially considering the lack of any non-overlapping quantitative morphological character to justify this separation.

Now, with all this said and done, I revisit the initial question posed to myself: where do I lie on the spectrum of lumpers vs. splitters? Considering my conclusions in the paragraph above, I think me a lumper – at least in the case of *Pediomelum*. And yet, my initial inclination – prior to this analytical undertaking – was to synonymize both *Pediomelum verdiense* and *Pediomelum pauperitense* under *Pediomelum mephiticum*. This exercise convinced me to do otherwise – to recognize *Pediomelum verdiense* at the species level. This is more leaning towards a splitter mentality. The problem? Not knowing the dimensions of the spectrum! I guess I lie somewhere in the middle…

## Conclusions

Given the conglomeration of past research with current findings shown herein, I support the recognition of *Pediomelum megalanthum* as having varieties *megalanthum* and *retrorsum*. I also recognize the specific status of *Pediomelum mephiticum*. As per the sinking of *Pediomelum pauperitense* under *Pediomelum verdiense*, a new description of *Pediomelum verdiense* is given below, along with a key to the taxa investigated or discussed herein.

### Key to the species

**Table:** 

1	Calyx tube less than 5.5 mm long	**2**
2	Bracts (7–)8–12.5 mm long; calyx tube 2.5–4 mm long; plants of sw UT, nw AZ, se NV	***Pediomelum mephiticum***
2'	Bracts (3–)4–8(–9) mm long; calyx tube (3.5–)4–5.5 mm long; plants of Mohave and Yavapai Cos, AZ	***Pediomelum verdiense***
1'	Calyx tube more than 5.5 mm long	**3**
3	Bracts caudate, 13–18×6–9 mm; Upper surfaces of leaflets glabrous to pubescent only along base of veins	***Pediomelum epipsilum***
3'	Bracts not caudate, or if caudate, not as large, 5–7×2.5–6 mm; Upper surfaces of leaflets pubescent throughout	**4**
4	Peduncle hairs shorter, appressed to incurved-ascending hairs and longer erect ones or sometimes with sparse, long curly hairs going in all directions	**Pediomelum megalanthum var. megalanthum**
4'	Peduncle hairs mostly long straight erect or reflexed hairs, or rarely of short and long hairs, but then both erect	**Pediomelum megalanthum var. retrorsum**

### *Pediomelum verdiense* S.L.Welsh & Licher

Western North American Naturalist 70: 12 (2010). *Type*: USA, Arizona, Yavapai Co., on the flats above a wash just north of Middle Verde exit from I-17, 18 April 2008, M. Licher 1911 (holotype BRY; isotype ASC).

*Pediomelum pauperitense* S.L.Welsh, Licher, & N.D.Atwood, Western North American Naturalist 70: 14 (2010). *Type*: USA, Arizona, Mohave Co., SW of Poverty Mountain, near Dewdrop Spring, 25 May 2001, L.C. Higgins 23135 (holotype BRY; isotypes distributed previously as *Pediomelum mephiticum*).

**Plant** acaulescent to short caulescent, 4.5–13(–15) cm tall, essentially glandular and pubescent throughout, from underground caudex branches arising from a deep, tuberous root. **Stems** 0–4(–6) cm, spreading white hairy; pseudoscapes 0–3, up to 6 cm, mainly subterranean; cataphylls 0–5 mm, glabrous to pubescent. **Leaves** clustered, palmately (3)5-foliolate; petioles 2–10(–11.5) cm long, with hairs appressed-ascending, jointed basally; stipules lanceolate to elliptic, scarious, 4–16 × 2–6 mm, tardily deciduous to persistent; petiolules 2–3 mm, pubescent; leaflet blades cuneate-obovate, (0.8–)1.2–3 × 0.7–1.8(–2.2) cm, cuneate basally, broadly acute to rounded or retuse apically, glandular and pubescent with more hairs along veins above and on lower surface, gray-green below, green to yellow-green above. **Inflorescence** globose, 1.5–3 cm long, with (1–)2–4(–6) nodes and (2)3-flowers per node; peduncles 0.5–4.5(–6) cm long, shorter than the petioles, spreading or spreading-ascending white-hairy, sometimes with longer spreading white hairs; bracts tardily deciduous to persistent, elliptic, 3.5–8.5(–10) × 2–6 mm. **Pedicels** filiform, 2.5–4.5(–6) mm long. **Flowers** (8–)10–13.5(–15) mm long, calyx (7.5–)8.5–12(–13) mm long, calyx-tube (2.5–)3.5–5 mm long, glandular, teeth lanceolate to oblong or elliptic, upper teeth 4–7(–8) × 1–2.5 mm, lower tooth (4–)5–9 × (1.5–)2.0–3.5 mm, gibbose-campanulate in fruit; petals white to purple, the banner white, cream, purple or suffused with pale purple, the wings and keel dark purple, with the wings sometimes lighter in color; 9–12(–14) × 6–8 mm with claw 2–5 mm, wings 10–13 × 2–3 mm with claw 4–5 mm, keel 8–10 × 2–3.5 mm with claw 3–5 mm; filaments 7–8.5 mm; anthers elliptic, 0.33 mm; ovary glabrous to pubescent apically, style concomitantly so basally. **Fruits** pubescent, eglandular, round to ovoid, body 5–7 × 3.5–5 mm, beak 1–4 mm, not exerted beyond calyx. **Seeds** oval to reniform, 3.5–5 mm × 2.5–3 mm, olive to gray brown and with or without purple mottling.

Flowering spring to summer. On limestone soils of the Verde Formation in Yavapai Co. and near Poverty Mountain in Mohave Co, Arizona.
